# Rebuilding life after breast cancer treatment among Korean breast cancer survivors: an integrative review

**DOI:** 10.4069/whn.2026.06.04

**Published:** 2026-06-30

**Authors:** Rina Kim, So-Young Choi

**Affiliations:** 1 Department of Nursing, Gyeongsang National University Hospital, Jinju, Korea; 2 College of Nursing, Gyeongsang National University, Jinju, Korea; 3 Sustainable Health Research Institute, Gyeongsang National University, Jinju, Korea

**Keywords:** Activities of daily living, Breast neoplasms, Cancer survivors, Psychological adaptation

## Abstract

**Purpose:**

This integrative review examined posttreatment experiences among Korean breast cancer survivors and synthesized the findings within a life-reconstitution framework.

**Methods:**

Whittemore and Knafl’s integrative review methodology was followed. Korean and international databases were searched from inception through January 19, 2026. Twelve publications met the inclusion criteria. Empirical studies were appraised using the 2018 Mixed Methods Appraisal Tool, and findings were synthesized through constant comparison. Reporting was guided by the Preferred Reporting Items for Systematic Reviews and Meta-Analyses 2020 statement.

**Results:**

Most included publications used cross-sectional designs and reported survivorship outcomes as separate variables rather than as components of a connected process. Four interrelated domains were identified: survivorship context, physical and functional constraints, psychological adjustment and role reintegration, and modifiable domains for survivorship care. Persistent physical problems, including pain, sleep disturbance, and activity limitations, formed the functional context of survivorship. Psychological concerns, including body-image concerns and fear of cancer recurrence, together with return to work, were central to posttreatment adjustment. Exercise, diet, self-management, and surveillance support were identified as potentially modifiable areas.

**Conclusion:**

Posttreatment survivorship among Korean breast cancer survivors can be understood as a process of rebuilding everyday life while managing persistent physical and psychosocial challenges. Integrating previous findings within a life-reconstitution framework provides a process-oriented perspective for survivorship research and care.

## Introduction

Advances in early diagnosis and treatment have substantially improved survival among patients with breast cancer. In South Korea, the 5-year relative survival rate for breast cancer during 2019 to 2023 was reported as 94.7% [1]. Breast cancer in Asian women generally occurs approximately 10 years earlier than in Western populations [2]. According to 2021 Korean national statistics, the median age at diagnosis among women with breast cancer was 53.4 years, and incidence was highest in the 40–49-year age group [3]. These epidemiological patterns indicate that cancer care should extend beyond survival to address posttreatment quality of life, continuity of care, and long-term management [4].

After treatment, breast cancer survivors frequently experience persistent physical and functional problems, including sleep disturbance, upper-extremity dysfunction, and breast cancer-related lymphedema; these problems are associated with poorer quality of life [5,6]. These sequelae extend beyond symptom burden and may restrict daily functioning and social participation. In an International Classification of Functioning, Disability and Health (ICF)-based study of Korean breast cancer survivors, impairments such as pain, sleep disturbance, decreased muscle strength, and lymphatic dysfunction were reported alongside restrictions in domestic, occupational, and leisure activities [7]. Accordingly, posttreatment physical changes and functional constraints provide an important context for understanding how breast cancer survivors rebuild everyday life.

Posttreatment experiences are also closely related to psychological and social processes. Distress related to altered appearance and body-image concerns can persist after treatment [8], and fear of cancer recurrence is one of the most common and clinically significant psychological challenges reported by breast cancer survivors [9]. Resuming daily and social roles is another important task during survivorship. Return to work, in particular, has repeatedly been described as a major posttreatment survivorship milestone, with fatigue, sleep disturbance, and psychological characteristics reported as associated factors [10]. These findings indicate that posttreatment life should be understood by considering physical problems, psychological adjustment, and role reintegration together.

A focused review of Korean breast cancer survivors is warranted because posttreatment experiences are shaped by sociocultural contexts, including social roles, family expectations, employment structures, and the healthcare system [2]. In this context, return to work has been consistently emphasized as a survivorship issue that reflects quality of life and social recovery among breast cancer survivors [10]. Some previous studies have also reported that long-term survivors adapt to a new daily life by reevaluating life priorities and meaning [11]. A comprehensive review of posttreatment experiences among Korean breast cancer survivors therefore does more than restrict the population to one country; it helps clarify how survivorship experiences have been formed and interpreted within a specific sociocultural context.

However, previous Korean reviews have mainly summarized individual outcome variables, such as quality of life, symptoms, and function [12,13]. Although this approach has identified factors and outcomes related to specific variables, it provides limited insight into how posttreatment survivorship unfolds over time or how physical and functional constraints, psychological adjustment, and role reintegration are related. Survivorship experiences therefore need to be interpreted not only as a list of symptoms and associated factors but also as a process through which life is reorganized after treatment. This perspective is important for establishing a conceptual foundation for evaluation frameworks and survivorship nursing interventions tailored to Korean breast cancer survivors.

In this study, a life-reconstitution perspective was used as the theoretical framework for interpreting posttreatment experiences among Korean breast cancer survivors. The chronic illness trajectory perspective helps explain why tasks such as symptom management, functional recovery, and surveillance support continue after treatment and why survivors adjust daily life while living with an altered health status [14]. The biographical disruption perspective emphasizes that cancer diagnosis and treatment can disrupt continuity in life, roles, and identity, requiring survivors to reinterpret and reorganize their lives [15]. The illness narrative perspective further helps explain how survivors make meaning of illness within their lives and develop new interpretations of altered bodies and daily routines [16]. In this review, life reconstitution was therefore used as an analytic framework for integrating posttreatment experiences—previously reported as separate outcome variables—into an interconnected adaptive process involving physical and functional constraints, psychological adjustment, role reintegration, modifiable domains, and intervention targets.

Accordingly, this integrative review aimed to examine how posttreatment experiences among Korean breast cancer survivors have been reported and conceptualized in the existing literature and to synthesize the findings through a life-reconstitution lens. By doing so, the review sought to identify core domains involved in rebuilding everyday life after treatment and to provide a conceptual foundation for survivor-centered nursing research and future intervention development.

## Methods

**Ethics statement:** This integrative review analyzed previously published literature and did not involve human participants, identifiable personal data, or direct data collection. Therefore, Institutional Review Board approval and informed consent were not required.

### Study design

**Figure 1.**
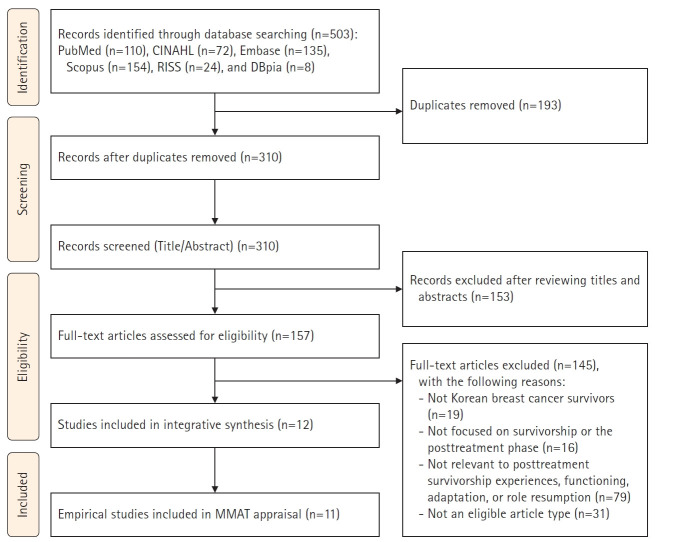
Flow diagram of the study process. MMAT: Mixed Methods Appraisal Tool.

An integrative review design was used to synthesize empirical and nonempirical literature addressing posttreatment experiences among Korean breast cancer survivors. The review followed the integrative review process proposed by Whittemore and Knafl [17]: problem identification, literature search, data evaluation, data analysis, and presentation of findings. Accordingly, this Methods section first presents the research problem and eligibility criteria, followed by the literature search, study selection, quality appraisal, and data analysis and synthesis procedures. To improve transparency in study identification and selection, the search and screening processes were reported with reference to the Preferred Reporting Items for Systematic Reviews and Meta-Analyses (PRISMA) 2020 statement, and the study selection process is presented in a flow diagram (Figure 1) [18].

### Problem identification and eligibility criteria

In the problem-identification stage, the primary analytic focus of this review was defined as posttreatment survivorship among Korean breast cancer survivors. The inclusion criteria were as follows. First, the study population had to consist of Korean breast cancer survivors; in multinational studies, publications were eligible only when data for the Korean subset were reported separately. Second, studies had to address functional recovery, life changes, adjustment, or posttreatment experiences among breast cancer survivors, including physical, psychological, or social role-related experiences. Third, eligible designs included qualitative, quantitative, mixed-methods, and intervention studies. Fourth, eligible publication types included peer-reviewed journal articles, dissertations with accessible full texts that permitted methodological appraisal, and academic book chapters with clear conceptual contributions and accessible full texts.

Studies covering both the active treatment and posttreatment phases were included if they reported findings directly related to rebuilding everyday life or readjusting to life after treatment. Studies focused exclusively on active treatment were excluded when they did not report posttreatment experiences. Publications were also excluded if they were not directly relevant to functional recovery or posttreatment experiences; were available only as abstracts, editorials, conference presentations, or reports without accessible full texts; or did not provide sufficient information to judge methodological quality or conceptual contribution. In this review, nonempirical literature referred to narrative reviews or academic book chapters that did not directly collect or analyze participant data; the term was not used synonymously with gray literature. Gray literature was not excluded categorically, and dissertations were included when full texts were available and methodological evaluation was possible. Abstracts, editorials, conference presentations, reports without accessible full texts, and materials unsuitable for methodological evaluation were excluded.

### Literature search

A comprehensive literature search was conducted to identify publications addressing posttreatment experiences and functional recovery among Korean breast cancer survivors. PubMed, Embase, CINAHL, and Scopus were searched as international databases, and RISS and DBpia were searched as Korean databases. The search period extended from the inception of each database through January 19, 2026.

The search strategy combined controlled vocabulary terms (MeSH/Emtree) with free-text terms, and search strings were adapted to the indexing system of each database. The core search concepts were (1) breast cancer (“Breast Neoplasms” or breast cancer), (2) survival and posttreatment phase terms (survivor, survivorship, posttreatment), (3) functional recovery and life change terms (function, functional recovery, activities of daily living, adaptation, adjustment, coping, transition, life change, lived experience, and related terms), and (4) Korea-related terms (Korea, Korean). These terms were combined using Boolean operators (AND, OR). For example, the following strategy was used in PubMed: (“Breast Neoplasms”[MeSH Terms] OR breast cancer*[Title/Abstract] OR breast neoplasm*[Title/Abstract]) AND (“Survivors”[MeSH Terms] OR survivor*[Title/Abstract] OR survivorship[Title/Abstract] OR post-treatment[Title/Abstract] OR posttreatment[Title/Abstract] OR “after treatment”[Title/Abstract]) AND (“functional recovery”[Title/Abstract] OR function*[Title/Abstract] OR rehabilitation[Title/Abstract] OR “Activities of Daily Living”[MeSH Terms] OR ADL[Title/Abstract] OR IADL[Title/Abstract] OR “physical function”[Title/Abstract] OR “role function*”[Title/Abstract] OR adaptation[Title/Abstract] OR adjustment[Title/Abstract] OR coping[Title/Abstract] OR transition*[Title/Abstract] OR “life change*”[Title/Abstract] OR “lived experience*”[Title/Abstract] OR normalization[Title/Abstract] OR “quality of life”[MeSH Terms]) AND (“Korea”[MeSH Terms] OR korea*[Title/Abstract] OR korean*[Title/Abstract] OR “South Korea”[Title/Abstract] OR “Republic of Korea”[Title/Abstract]). Detailed search queries and the number of results for each database are provided in the Supplementary Data.

### Study selection

Retrieved records were imported into EndNote (Clarivate, Philadelphia, PA, USA), and duplicates were removed. Title and abstract screening was then conducted according to the predefined eligibility criteria, followed by full-text review of potentially relevant publications. The researcher and a doctoral-level nursing researcher independently performed both title/abstract screening and full-text review. Discrepancies between the two reviewers were resolved through discussion and reexamination of the eligibility criteria until consensus was reached.

A total of 503 records were identified. After duplicates were removed, 310 records underwent title and abstract screening. Of these, 157 full texts were assessed for eligibility, and 12 publications were ultimately included. The final selection process and reasons for full-text exclusion are presented in the flow diagram (Figure 1) [18].

### Data evaluation

Methodological quality was appraised for the 11 empirical studies among the 12 included publications; the single nonempirical publication was excluded from appraisal. The 2018 Mixed Methods Appraisal Tool (MMAT) was used. Screening questions were first applied to confirm appraisal suitability, after which studies were rated as “Yes,” “No,” or “Can’t tell” according to the MMAT criteria for each study design. The researcher and a doctoral-level nursing researcher independently conducted the quality appraisal, and discrepancies were resolved through discussion.

Because the MMAT is designed to appraise empirical studies, the nonempirical publication was not evaluated with this tool. However, it was retained in the synthesis because it was judged to contribute to conceptual integration. A rating of “Can’t tell” does not necessarily indicate low methodological quality; rather, it indicates that judgment was limited by insufficient reporting. Quality appraisal was not used as an exclusion criterion. Instead, appraisal results were used during synthesis to consider methodological limitations and reporting adequacy when interpreting findings. No analytic category was derived from a single study alone; greater interpretive weight was assigned to patterns repeatedly observed across multiple study designs and data sources.

### Data analysis and synthesis

During data analysis and synthesis, information was extracted on study characteristics, survivorship stage, conceptual definitions, measurement methods, and major findings related to functional recovery and posttreatment experiences. The extracted findings were organized into meaning units. Iterative within-study and across-study comparisons were then conducted to identify commonalities, differences, and recurrent patterns. Constant comparison was applied throughout the analysis. Findings with similar meanings were synthesized into higher-order categories, and relationships among categories were examined iteratively.

The categories derived from this process were synthesized into four domains: (1) survivorship context, (2) physical and functional constraints, (3) psychological adjustment and role reintegration, and (4) modifiable domains and intervention targets. Finally, these domains were interpreted through a life-reconstitution lens to synthesize posttreatment survivorship as an interconnected adaptive process rather than as a disconnected list of individual outcome variables.

### Presentation of findings

**Figure 2.**
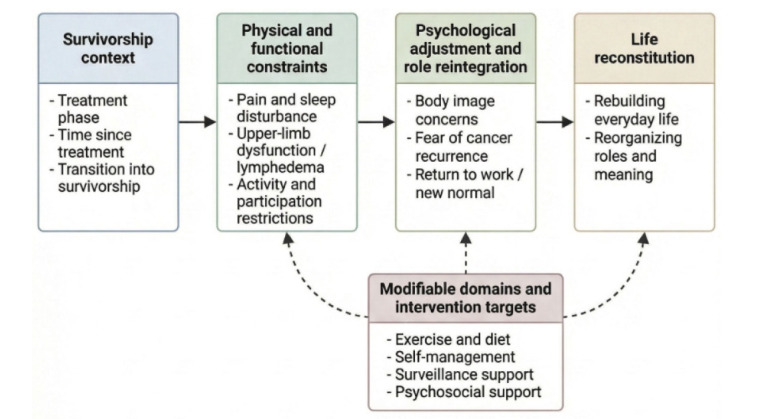
Proposed framework of life reconstitution as an interrelated posttreatment survivorship process among Korean breast cancer survivors.

The methodological appraisal results for the included empirical studies are summarized in Table 1 [7-11,19-24]. The characteristics of each publication and its primary analytic contribution to the life-reconstitution process are presented in Table 2 [7-11,19-25]. The study selection process is shown in Figure 1, and the integrative conceptual model of life reconstitution among Korean breast cancer survivors is shown in Figure 2. Abbreviations used in each table and figure are defined in the corresponding footnotes.

## Results

### Overview of included publications and process-oriented synthesis

A total of 12 publications were included. Most used cross-sectional designs; the review also included prospective cohort studies, a pilot randomized controlled trial, qualitative studies, and one nonempirical publication. Overall, the included studies tended to report individual outcome variables—such as symptoms, psychological status, return to work, self-management, and surveillance—rather than presenting posttreatment survivorship as a temporally connected adaptation process.

In this synthesis, recurrent findings across studies were compared and integrated into four domains: (1) survivorship context, (2) physical and functional constraints, (3) psychological adjustment and role reintegration, and (4) modifiable domains and intervention targets. Survivorship context captured the temporal and clinical background in which posttreatment experiences were reported. Physical and functional constraints encompassed persistent physical symptoms and activity limitations after treatment. Psychological adjustment and role reintegration included body-image concerns, fear of cancer recurrence, return to work, and formation of a new normal. Modifiable domains and intervention targets included intervention-related findings that may support posttreatment adjustment, such as exercise, diet, self-management, and surveillance support. The characteristics of the included publications and their positions within the life-reconstitution framework are presented in Table 2, and the overall conceptual model is shown in Figure 2.

### Survivorship context

The included studies covered diverse survivorship stages, ranging from active treatment to early and long-term survivorship. However, social and environmental contextual factors beyond the treatment phase were not measured consistently, and most studies reported outcomes at specific time points. Therefore, in this synthesis, survivorship context was structured primarily around treatment phase and time since treatment completion. This domain was used to summarize the temporal background in which the included findings were reported.

Some studies included participants during and after treatment [8]. Other studies focused on survivors at specific posttreatment intervals or on long-term survivors [10,11]. Research on surveillance support targeted survivors who were at least 1 year posttreatment [24]. A prospective cohort study examined changes from the preoperative period to 12 months after surgery [19]. A study using National Health Insurance data presented prescription patterns according to survivorship duration [20]. Thus, survivorship stages and observation time points differed across the included studies, and this synthesis considered those differences as contextual background for integrating subsequent findings.

### Physical and functional constraints

Physical symptoms and functional limitations were reported repeatedly in studies of posttreatment survivorship. An ICF-based functional assessment study reported impairments such as pain, sleep disturbance, decreased muscle strength, reduced exercise tolerance, and lymphatic dysfunction. These impairments were accompanied by restrictions in activity and participation domains, including domestic life, occupational activities, leisure, and maintenance of daily routines [7]. A prospective cohort study indicated that although some domains of sexual concerns partially improved over time, concerns related to sexual interest and satisfaction could persist; upper-extremity dysfunction and lymphedema were associated with these concerns [19]. A National Health Insurance cohort study also showed that prescriptions for gastrointestinal medications, calcium supplements, and anxiolytics persisted across survivorship [20]. Physical and functional constraints were therefore interpreted as persistent conditions that may require survivors to adjust daily activities and social participation after treatment.

### Psychological adjustment and role reintegration

Findings related to psychological adjustment and role reintegration were also reported repeatedly across the included studies. Distress related to altered appearance and body-image concerns was documented both during and after treatment [8]. Fear of cancer recurrence emerged as a recurring psychological factor during survivorship and was associated with negative psychosocial outcomes [9]. Age-related differences in psychosocial adjustment were also reported, suggesting that adjustment patterns may vary by life stage [21].

Regarding role reintegration, return to work was a key outcome. Related studies reported that fatigue, sleep disturbance, and psychological characteristics were associated with return to work [10]. A qualitative study described how survivors transitioned to a new normal by reorganizing life priorities and meaning [11]. Another qualitative study reported that the trajectory from diagnosis through treatment and recovery to return to work could differ by life stage [22].

In this synthesis, outcomes related to body-image changes, fear of cancer recurrence, age-related differences in psychosocial adjustment, return to work, and formation of a new normal were consolidated into the domain of psychological adjustment and role reintegration. This domain encompassed findings in which posttreatment emotional responses and recovery of social roles were reported together.

### Modifiable domains and intervention targets

Several studies reported modifiable domains that may support posttreatment adjustment. A phase-tailored exercise and dietary intervention study reported feasibility and improvements in emotional functioning and fatigue [23]. In a study of surveillance support, depressive symptoms and psychosocial barriers were associated with lower adherence to mammography screening [24]. Literature addressing diet- and metabolism-related factors also proposed support strategies for lifestyle management [25].

In this synthesis, exercise, diet, self-management, and surveillance support were categorized as modifiable domains in posttreatment care. These categories were framed not as direct outcomes of posttreatment survivorship, but as practical intervention targets for survivorship nursing care and intervention development.

### Integrated interpretation of the four domains

The four domains derived from this synthesis were structured not as a parallel list of outcomes but as interrelated layers of posttreatment survivorship. Survivorship context served as the background domain, describing treatment phase and time since treatment completion across the included studies. Physical and functional constraints encompassed repeatedly reported physical symptoms and functional limitations after treatment. Psychological adjustment and role reintegration included outcomes related to body-image concerns, fear of cancer recurrence, return to work, and establishment of a new normal. Modifiable domains and intervention targets were organized as areas that may support posttreatment care, including exercise, diet, self-management, and surveillance support. Together, this synthesis reinterprets posttreatment experiences that previous studies reported as isolated outcome variables through the lens of life reconstitution.

## Discussion

This integrative review shows that posttreatment life among Korean breast cancer survivors is not simply a linear recovery process marked by symptom reduction or functional restoration. Rather, it is an adaptive process in which survivors reorganize everyday life, roles, and life meaning while managing persistent physical and functional constraints and psychosocial demands. The four domains derived from this review can be understood not as independent outcome categories, but as interconnected elements that constitute the context, conditions, adaptive processes, and intervention points through which posttreatment survivorship unfolds. Unlike previous studies that have reported posttreatment experiences as isolated outcomes, such as symptoms, quality of life, psychological states, or return to work, these findings suggest the need to interpret such outcomes within a broader process of life reconstitution [10,12]. The main contribution of this review is therefore not the addition of new individual variables, but the organization of previously fragmented findings into a process-oriented structure of posttreatment life reconstitution.

The focus on Korean breast cancer survivors is meaningful not merely as a narrowing of scope, but as an analytic choice based on the premise that posttreatment survivorship is shaped by sociocultural context. Although breast cancer survivorship involves medical challenges shared across countries [4], posttreatment life is also influenced by family roles, occupational structures, social expectations, healthcare access, and follow-up care practices. International literature has consistently reported physical symptoms, functional decline, and quality of life concerns as major survivorship issues [5,6], which is consistent with the physical and functional constraints identified in this review. Breast cancer incidence among Asian women has also been reported to peak at a younger age than in Western populations [2], providing context for the importance of return to work and family and social role reintegration in the present synthesis. In the Korean literature included in this review, distress related to altered appearance and body-image concerns was reported as a posttreatment adjustment issue [8], and fear of cancer recurrence was identified as a major psychological challenge [9]. Return to work was repeatedly reported as an important survivorship issue in studies of Korean breast cancer survivors [10,22]. Qualitative studies also described long-term survivors’ experiences of moving toward a new normal by reorganizing life priorities and meaning [11]. These findings do not imply that Korean breast cancer survivors’ experiences are entirely distinct from those of survivors in other countries. Rather, they indicate that survivorship should be interpreted within the specific contexts of age at diagnosis, family and social role expectations, employment structures, and follow-up care practices in Korea.

The first pattern identified in this review was that physical and functional constraints persisted after treatment and served as background conditions for everyday survivorship. A Korean ICF-based study reported that pain, sleep disturbance, decreased muscle strength, and lymphatic dysfunction were linked to activity and participation restrictions [7]. A prospective cohort study indicated that some domains of sexual concerns may persist over time and that upper-extremity dysfunction and lymphedema were associated with these concerns [19]. Research using National Health Insurance data also identified patterns of medication use across survivorship [20]. These findings indicate that posttreatment problems should not be understood only as a list of sequelae; rather, they should be interpreted as continuing conditions that shape survivors’ daily activities, social participation, and self-management. This interpretation is consistent with international literature reporting that sleep disturbance, upper-extremity dysfunction, and lymphedema among breast cancer survivors are associated with poorer quality of life [5,6]. Thus, physical and functional problems such as pain, sleep disturbance, upper-extremity dysfunction, and lymphedema may be understood as persistent factors that influence how survivors adjust daily routines, cope with psychological burden, and return to social roles.

The second pattern was that psychological adjustment and role reintegration were central to the direction of posttreatment life. Distress related to altered appearance and body-image concerns could persist after treatment completion [8], and fear of cancer recurrence was identified as a prominent psychological burden [9]. These psychological issues are not limited to emotional responses; they are also connected to how survivors reinterpret their bodies, health, futures, and everyday roles. Return to work was repeatedly highlighted in Korean studies as an important survivorship milestone [10,22], closely related to survivors’ resumption of social roles and agency in daily life. The transition to a new normal, as reported in qualitative research [11], can be understood in the same context. Given the relatively younger age at diagnosis among Korean women with breast cancer and the potential overlap of occupational, family, and caregiving roles, return to work and role reintegration provide important contextual points for explaining life reconstitution after treatment.

Third, this review identified modifiable domains and intervention targets that may support survivors’ adjustment after treatment. A phase-tailored exercise and dietary intervention was associated with improvements in fatigue and emotional functioning [23], and a study of surveillance adherence showed that depressive symptoms and psychosocial barriers were associated with lower adherence to surveillance mammography [24]. Literature on diet- and metabolism-related factors also emphasized the importance of lifestyle management [25]. These findings suggest that exercise, diet, self-management, follow-up care, and psychosocial support are not merely separate intervention topics; they may represent key areas of survivorship nursing that support posttreatment adjustment. However, because the current evidence comes mainly from observational studies and small-scale intervention trials, the extent to which these domains change long-term survivorship trajectories remains insufficiently established. Nevertheless, this review frames modifiable domains and intervention targets not only as health-behavior recommendations but also as practical targets for supporting life reconstitution.

From a theoretical perspective, this review applied the life-reconstitution perspective introduced in the Introduction to an empirical literature synthesis. In doing so, it connected physical and functional constraints, psychological adjustment, role reintegration, and modifiable domains and intervention targets within a single process structure. The chronic illness trajectory perspective was useful for understanding the ongoing management burden and adjustment process that continues after treatment [14]. The biographical disruption perspective supported interpretation of body-image changes, fear of cancer recurrence, and return-to-work experiences as part of a process in which life continuity is disrupted [15]. The illness narrative perspective provided a theoretical basis for understanding survivors’ reconstruction of daily life and life meaning [16].

This review also differs analytically from previous Korean review studies. Earlier reviews helped summarize factors related to quality of life and the direction and magnitude of associated variables [12,13], but they did not fully show how posttreatment experiences unfold over time, how physical and functional constraints are related to psychological adjustment and role reintegration, or where nursing interventions may be positioned within a unified structure. This review addresses that gap by reinterpreting posttreatment survivorship among Korean breast cancer survivors as a process-oriented concept. However, it does not empirically confirm phenomena that are unique to Korean survivors. Its significance lies in organizing recurrent findings from Korean literature into a framework that can be interpreted within a sociocultural context. This review may therefore serve as a conceptual starting point for future studies that measure and explain survivorship experiences and for intervention-development research aimed at supporting posttreatment adjustment.

From a practice perspective, these findings suggest that symptom management alone is insufficient for understanding posttreatment life among breast cancer survivors; long-term survivorship nursing should also address functional recovery, psychological adjustment, and role reintegration. First, nurses should assess persistent physical problems such as pain, sleep disturbance, upper-extremity dysfunction, and lymphedema not only as sequelae but also as conditions that restrict daily functioning and social participation [7,19]. Second, because fear of cancer recurrence and body-image concerns can persist after treatment completion, early screening for emotional distress and linkage to appropriate counseling, education, and support systems are needed [8,9]. Third, return to work and role reintegration may serve as important recovery indicators during survivorship; therefore, nursing interventions should assess and support occupational return, changes in family roles, and social adjustment together [10,22]. Fourth, exercise, diet, self-management, and surveillance support may be considered modifiable domains and intervention targets that can support survivorship care [23,24]. Literature on diet- and metabolism-related factors also supports this direction [25]. Future survivorship nursing programs for breast cancer survivors should therefore move beyond physical symptom monitoring and integrate functional recovery, emotional support, role reintegration, self-management, and surveillance support.

This review has several limitations. First, most included studies used cross-sectional designs, limiting the ability to determine temporal ordering or causal directions among physical and functional constraints, psychological adjustment, and role reintegration. Second, definitions of survivorship and measurement time points varied across studies, limiting direct comparison of posttreatment experiences. Third, although this review focused on literature concerning Korean breast cancer survivors, few studies directly measured or analyzed sociocultural contextual factors. Therefore, the findings should not be interpreted as characteristics unique to Korean survivors. Fourth, because the included studies were not designed for cross-national comparison or direct testing of cultural factors, they cannot empirically establish Korean specificity. Fifth, the number of included publications was relatively small, and the inclusion of dissertations and nonempirical literature limited the breadth of the evidence base. Sixth, because the synthesis relied on published literature and accessible materials, publication bias cannot be excluded. Finally, although methodological quality appraisal was used as a reference for interpretation, some included studies had limited reporting; therefore, some findings should be interpreted cautiously.

Future research should prioritize longitudinal designs to clarify adjustment trajectories from immediate treatment completion through long-term survivorship. In particular, studies are needed to examine how physical and functional constraints change over time and how these changes are related to fear of cancer recurrence, body image, role reintegration, and return to work. Mixed-methods research may also help explain meaning reconstruction, restoration of everyday life, and role negotiation processes that are difficult to capture with quantitative indicators alone. Comparative studies are needed to examine how family roles, occupational return, body-image experiences, and follow-up care practices among Korean breast cancer survivors are similar to or different from those of survivors in other sociocultural settings. In addition, measurement tools based on the core domains identified in this review should be developed and validated to assess life reconstitution among Korean breast cancer survivors. Finally, future studies should develop integrated survivorship interventions that combine exercise, diet, self-management, psychosocial support, and follow-up care support and test whether these interventions improve posttreatment adjustment and life reconstitution.

In summary, posttreatment life among Korean breast cancer survivors can be understood not as the simple completion of recovery but as a process of rebuilding everyday life through psychological adjustment and role reintegration while managing persistent physical and functional constraints. This review has theoretical significance because it reinterprets symptoms, psychosocial burden, role recovery, self-management, and follow-up care findings that have often been reported separately within a process-oriented framework of life reconstitution. The synthesis also suggests that breast cancer survivorship nursing should extend beyond symptom monitoring to include long-term support for functional recovery, emotional well-being, role reintegration, self-management, and follow-up care. This review therefore provides a conceptual foundation for future research on explaining and evaluating posttreatment life among Korean breast cancer survivors and for developing survivorship nursing interventions.

## Figures and Tables

**Table 1.** Methodological appraisal of empirical studies included in the integrative review using Mixed Methods Appraisal Tool (MMAT) (2018) (N=11)

Studies	Design	MMAT categories	Yes (n)	No (n)	Can’t tell (n)
Kang et al. [8]	Cross-sectional	Quantitative, non-randomized	3	0	2
Sohn et al. [10]	Cross-sectional	Quantitative, non-randomized	3	0	2
Lee et al. [9]	Cross-sectional	Quantitative, non-randomized	3	0	2
Jang et al. [24]	Cross-sectional	Quantitative, non-randomized	3	0	2
Kim and Ko [21]	Cross-sectional	Quantitative, non-randomized	3	0	2
Oh et al. [20]	Retrospective cohort	Quantitative, non-randomized	3	0	2
Yang et al. [7]	Instrument development and cross-sectional	Quantitative, descriptive	3	0	2
Yang et al. [19]	Prospective cohort	Quantitative, non-randomized	4	0	1
Kim et al. [23]	Pilot randomized controlled trial	Quantitative, randomized	3	0	2
Baek [11]	Qualitative	Qualitative	4	0	1
Bong [22]	Qualitative thesis	Qualitative	3	0	2

**Table 2.** Characteristics of included publications and their primary contribution to the life-reconstitution process (N=12)

Studies	Design	Survivorship phases	Primary analytic contribution	Key focus
Kang et al. [8]	Cross-sectional	During and after treatment	Psychological adjustment and role reintegration	Body-image distress; altered appearance
Kim et al. [23]	Pilot randomized controlled trial	Posttreatment	Modifiable domains and intervention targets	Exercise and diet intervention; emotional functioning; fatigue
Lee et al. [9]	Cross-sectional	Posttreatment	Psychological adjustment and role reintegration	Fear of cancer recurrence; psychosocial outcomes
Sohn et al. [10]	Cross-sectional	≤10 years after diagnosis	Psychological adjustment and role reintegration	Return to work; fatigue; work-related adjustment
Jang et al. [24]	Cross-sectional	≥1 year after treatment	Modifiable domains and intervention targets	Surveillance adherence; depressive symptoms
Oh et al. [20]	Retrospective cohort	<1 to ≥5 years of survivorship	Physical and functional constraints	Supportive medication use; ongoing management
Yang et al. [7]	Instrument development and cross-sectional	≤1 and ≥1 year after treatment	Physical and functional constraints	ICF-based activity and participation restrictions
Yang et al. [19]	Prospective cohort	Preoperative period to 12 months after surgery	Physical and functional constraints	Sexual concerns; upper-extremity dysfunction
Kim and Ko [21]	Cross-sectional	Posttreatment	Psychological adjustment and role reintegration	Age-related psychosocial adjustment differences
Baek [11]	Qualitative ethnography	≥5 years of survivorship	Psychological adjustment and role reintegration	Meaning reconstruction; new normal
Bong [22]	Qualitative thematic study	Long-term survivorship	Psychological adjustment and role reintegration	Diagnosis-to-return-to-work trajectory
Lee [25]	Narrative review, book chapter	Before and after diagnosis	Modifiable domains and intervention targets	Diet- and metabolism-related factors; supportive strategies

ICF: International Classification of Functioning, Disability and Health.

## Supplementary materials

Further details on supplementary materials are presented online (available at https://doi.org/10.4069/whn.2026.06.04).
