# Supplementary material for: Rebuilding life after breast cancer treatment among Korean breast cancer survivors: an integrative review
Source: Womens Health Nurs. 2026 Jun 30;32(2):104–15. doi: 10.4069/whn.2026.06.04 (PMC13346790; doi:10.4069/whn.2026.06.04)
Supplement: Supplementary Data. [file whn-2026-06-04-Supplementary-Data.pdf]

Supplementary Data

1. Overview of search process

A comprehensive literature search was conducted to identify studies addressing posttreatment functional recovery and life changes among Korean breast cancer survivors. Six electronic databases were searched: PubMed, Embase, CINAHL, and Scopus for international literature, and RISS and DBpia for domestic literature. Searches covered all available years in each database from inception to January 19, 2026. Medical Subject Headings (MeSH)/Emtree terms and free-text keywords were combined using Boolean operators (OR, AND), and the strategy was adapted to the indexing system of each database. Full search strategies and search yields by database are provided below to enhance transparency and reproducibility.

2. Database search strategies

The detailed search terms and the number of results for each database are presented in the tables below.

Search strategies by database (Search Date: 2026-01-19)

PICO framework

P	I	C	O
Korean breast cancer survivors	Cancer survivorship (posttreatment phase)	-	Functional recovery,life changes, psychosocial adaptation,role transition
